# Biocontrol of Seedborne Fungi on Small-Grained Cereals Using *Bacillus halotolerans* Strain B33

**DOI:** 10.3390/jof11020144

**Published:** 2025-02-13

**Authors:** Tatjana Popović Milovanović, Renata Iličić, Ferenc Bagi, Goran Aleksić, Nenad Trkulja, Vojislav Trkulja, Aleksandra Jelušić

**Affiliations:** 1 Institute for Plant Protection and Environment, Teodora Drajzera 9, 11040 Belgrade, Serbia; alegoran@gmail.com (G.A.); trkulja_nenad@yahoo.com (N.T.); 2 Faculty of Agriculture, University of Novi Sad, Trg Dositeja Obradovića 8, 21000 Novi Sad, Serbia; renatailicic@gmail.com (R.I.); bagifer@gmail.com (F.B.); 3 Agricultural Institute of Republic of Srpska, Knjaza Milosa 17, 78000 Banja Luka, Bosnia and Herzegovina; vtrkulja@blic.net; 4 Institute for Multidisciplinary Research, University of Belgrade, Kneza Višeslava 1, 11030 Belgrade, Serbia; jelusic.aleksandra@gmail.com

**Keywords:** *Fusarium graminearum*, *Alternaria alternata*, *Aspergillus flavus*, grains, biological control

## Abstract

The development of biological pesticides is rapidly becoming an integral aspect of pest management in sustainable agriculture. This study was conducted to evaluate the effectiveness of *Bacillus halotolerans* strain B33 against three common seedborne fungal pathogens—*Fusarium graminearum*, *Alternaria alternata*, and *Aspergillus flavus*. B33 strain identity was determined using the 16S rRNA and *tuf* gene sequences. Commercial wheat, barley, oat, and rye seeds were artificially infected by fungal isolates and then treated with B33 overnight culture in Nutrient Broth. The obtained results indicate high efficacy against *F. graminearum* (83.55–94.38%) and *A. alternata* (85.05–96.70%), whereby the highest efficacy was noted on wheat seed and the lowest was detected on rye seed. On the other hand, B33 achieved 100% efficacy against *A. flavus* on barley, rye, and oat seeds, while being 96.24% effective against this pathogen on wheat. Principal component analysis indicated the highest treatment influence on *A. flavus*. The effect of all tested treatments on seed germination was statistically significant compared to the controls, whereby the number of germinated seeds declined as the seed infection rate increased. *B. halotolerans* strain B33 effectively managed seedborne fungal pathogens, thereby enhancing seed germination.

## 1. Introduction

Small-grained cereals (family *Poaceae*), including wheats (*Triticum aestivum*, *T. spelta*, *T. durum*, *T. dicoccon*), triticale (x *Triticosecale* spp.), barley (*Hordeum vulgare*), oats (*Avena sativa*), and rye (*Secale cereale*), represent crop plants grown for their edible grain. Wheat is the most important cereal for human consumption, along with rice (*Oryza sativa* L.) and maize (*Zea mays* L.) [1]. Due to their high nutritional value provided by the constituent carbohydrates, proteins, vitamins, and minerals, as well as a range of phytochemicals providing health benefits, cereals are an indispensable component of the human diet [2]. Wheat straw represents the largest potential source of feedstock for the production of second-generation biofuels in Europe [3]. According to the latest available data from the Food and Agriculture Organization of the United Nations (FAOSTAT), 3,059,640,000 t of cereals was produced worldwide in 2022, with wheat, as a leading small-grained cereal, accounting for 808,442,000 thousand tones [4], while in Serbia, 3,448,700 t was produced in 2023 [5].

Current cereal production (wheat in particular) trends parallel global population growth and thus respond to the increasing demand for this valuable source of nutrition [6]. However, such intensive cereal production is threatened by a variety of biotic and abiotic stresses at all developmental stages. Seedborne fungal diseases are particularly important carriers of pathogens, allowing their long-distance spread and inter-regional survival, resulting in not only significant yield reduction (15–90%) by adversely impacting the seed germination rate and vigor but also lower commercial grain quality [7]. The presence of several fungal genera—*Alternaria*, *Aspergillus*, *Cladosporium*, *Curvularia*, *Fusarium*, *Helminthosporium*, *Penicillium*, *Rhizopus,* and *Stemphylium*—has been previously reported in cereal seeds [8]. These fungi are often very destructive if not controlled in time. Seed treatment is beneficial against seedborne pathogens, especially early in the growing season, as crops are most susceptible during their early growth, when their immune system is still underdeveloped.

Under the sustainable agriculture paradigm, chemical seed-applied pesticides are increasingly being replaced by biological agents as a means of seedborne pathogen control. *Bacillus* species are presently considered the most promising alternative to pesticide use due to their multifarious functional attributes [9]. Besides their biocontrol properties, they may also act as biostimulants owing to their ability to produce indole-3-acetic acid, fix nitrogen, etc. Given their exposure to more extreme or varied environmental conditions (temperature fluctuations, nutrient limitations, pH and humidity variability, etc.), non-agricultural soils serve as an excellent reservoir for isolating *Bacillus* strains with unique metabolic capabilities. Additionally, agricultural soils are subjected to prolonged and excessive pesticide application, and in some countries antibiotics are routinely used, resulting in the development of resistance in microorganisms. Therefore, soil bacteria, including *Bacillus* spp., may act as recipients and reservoirs of resistance genes originating from clinical, livestock, or agricultural sources [10]. The most common *Bacillus* species that are currently commercialized as biopesticides or fertilizers are *B. thuringiensis*, *B. subtilis*, *B. amyloliquefaciens*, *B. velezensis*, and *B. megaterium*. *Bacillus halotolerans* has been recently described as a potent agent against biotic and abiotic stress; however, its potential has not yet been commercially exploited.

Therefore, the objective of this study was to isolate and characterize promising antagonistic isolates from the genera *Bacillus* that could be potentially applied in the biological control of seedborne polyphagous fungi on small-grained cereals, chosen as a plant model system owing to their economic importance and worldwide distribution.

## 2. Materials and Methods

### 2.1. Seed Material and Seedborne Fungi

Seed material was collected in 2023 from the commercial cereal (wheat cultivar Simonida, barley cultivar Nonius, oat cultivar Sedef, and rye cultivar Savo) plots located in the Bačka region (Vojvodina, Serbia). All seeds used for this study were taken after classic harvesting and were stored in paper bags at 8–10 °C until required for analyses. One day before initiating the experiments, sample seeds were superficially disinfected with 1% sodium hypochlorite for 20 min, rinsed under tap water, and dried on filter paper at room temperature.

Strains of seedborne pathogenic fungi *Fusarium graminearum* and *Alternaria alternata* originating from wheat (coded as WFG09 and WAA6, respectively, sourced from the collection of T.P. Milovanović, Institute for Plant Protection and Environment), as well as *Aspergillus flavus*—a fungal pathogen of major economic and public health concern, causing aflatoxin contamination in maize and opportunistic infection in other cereal seeds (coded as BFF15, obtained from the collection of F. Bagi, Faculty of Agriculture)—were included in the experiments. Potato Dextrose Agar (PDA) containing Streptomycin (100 ppm) was used as the growth medium. Using a compound microscope (Olympus BX51TF, Olympus, Japan), all fungal strains were checked for the colony form and color, as well as the shape, color, and dimensions of spores [11,12,13].

### 2.2. Isolation, Selection, and Identification of Potential Antagonistic Bacteria

The isolation of the potential antagonistic bacteria was performed from five soil samples (around 10 g each), which were taken at 5–10 cm depths from a rural, non-agricultural area near the Danube River (Smederevo site). For this purpose, 1 g of soil from each sample was placed in a test tube containing 1 mL of Nutrient Broth (NB) and was incubated at a heating blot at 80 °C for 10 min to separate vegetative cells from endospores. Thereafter, the suspension was incubated at 30 °C overnight before being plated on the Luria–Bertani Agar (LA). All morphologically different *Bacillus*-like colonies that developed on each sample after 24 h incubation at 30 °C (17 in total) were selected, purified, and maintained in sterile LB glycerol stocks at −80 °C [14].

The dual culture assay was used to test the antagonistic activity of *Bacillus*-like isolates against seedborne fungal pathogens in vitro. Using a sterile needle, mycelium discs (measuring 5 mm in diameter) of the tested seedborne fungal strains (*F. graminearum*, *A. alternata,* and *A. flavus*) were inserted in the centers of individual Petri plates on PDA. Then, four 5 µL droplets of overnight-cultured *Bacillus*-like isolates adjusted to approximately 10^8^ CFU mL^−1^ were deposited on the 2 cm perimeter around the center. Inoculated Petri plates were kept at 25 °C and fungal growth was rated on a daily basis. Plates inoculated only with mycelia were used as the positive control (PC). The experiment was performed in four replicates. The inhibition zone was calculated according to the following formula:

$$\mathrm{Inhibition}\;(\%)=[\frac{Colony\;diameter\;of\;PC-Treated\;colony\;diameter}{Colony\;diameter\;of\;PC}]\;\times \;100$$

Using the assay described above, five isolates (coded as BML2, B8, B29, B30, and B33) with the most pronounced in vitro antagonistic activity were selected for molecular identification. DNA extraction from these isolates was performed using the CTAB method [15]. Isolates were identified by DNA amplification with two primer pairs—(i) P0 (5′-GAGAGTTTGATCCTGGCTCAG-3′)/P6 (5′-CTACGGCTACCTTGTTACGA-3′) [16] and (ii) tufGPF (5′-ACGTTGACTGCCCAGGACAC-3′)/tufGPR (5′-GATACCAGTTACGTCAGTTGTACGGA-3′) [17]—based on the partial sequences of 16S rRNA and elongation factor thermal unstable Tu (*tuf*) genes, respectively. PCR reactions were performed in a total reaction volume of 25 µL consisting of 12.5 µL of 2 × PCR TaqNova-RED Master Mix (DNA Gdansk, Gdańsk, Poland), 9.5 μL of PCR-grade water, 1 µL of each of the primers (10 μM), and 1 µL of sample DNA. Amplification conditions comprised initial denaturation at 95 °C for 8 min (*tuf*) or 10 min (16S rRNA), 30 (16S rRNA) or 35 (*tuf*) cycles of denaturation at 95 °C for 30 s (*tuf*) or 94 °C for 1 min (16S rRNA), annealing at 55 °C for 1 min (*tuf*) or 90 s (16S rRNA), extension at 72 °C for 30 s (*tuf*) or 150 s (16S rRNA), and a final elongation step at 72 °C for 10 min.

The obtained PCR products were sent for Sanger sequencing to Eurofins Genomics (München, Germany). Once received, the sequences were manually checked for quality and were compared with the sequences available in the National Center for Biotechnology Information (NCBI) database using the nucleotide BLAST (BLASTn) function. Only isolates belonging to the Risk 1 biosafety level group according to the German Technical Rules for Biological Agents (TRBA) classification [18] were subjected to phylogenetic analysis.

Phylogenetic analysis was performed with the concatenated sequences of both genes (16S rRNA and *tuf*) for the finally selected biocontrol candidate isolates and nine comparative *Bacillus* spp. strains (*B. amyloliquefaciens*, *B. halotolerans*, *B. safensis*, *B. subtilis*, and *B. velezensis*) retrieved from the GenBank database (Table 1). Before the construction of the neighbor-joining (NJ) phylogenetic tree, sequences of the tested and comparative isolates/strains were aligned in the BioEdit sequence alignment editor (v7.0) program and trimmed to the same size (2114 nt). The NJ tree was constructed in MEGA software (v7.0). *Gracilibacillus salitolerans* strain SCU50 (Acc. No. CP045915) from the NCBI was used as an outgroup strain.

Given that certain strains of *B. halotolerans* (coded as LDFZ001, KLBC XJ-5, JK-25, LBG-1-13, LYSX1, and QTH8 [19,20,21,22,23,24,25]) with previously demonstrated biocontrol ability in other settings were deposited to the GenBank database using partial 16S rRNA sequences (Table 1), another NJ tree was generated solely based on the relevant 16S rRNA sequences (1363 nt). The same outgroup strain as specified above was used to root this tree.

### 2.3. Seed Treatment with the Candidate Antagonistic Strain B33

Experiments were performed using a biocontrol candidate that exhibited the most pronounced in vitro antagonistic activity among all 17 initially screened isolates. The biocontrol ability of the selected isolate against three selected seedborne fungal isolates was assessed on wheat, barley, rye, and oat seeds. For the seed inoculation purposes, the selected fungal strains WFG09 and WAA6 (representing *F. graminearum* and *A. alternata*, respectively) and the *A. flavus* strain BFF15 were grown on Czapek Yeast Autolysate Agar at 25 °C in the dark for 10 days. Next, 100 mL of ddH_2_O was poured into each Petri plate, and conidia and hyphal fragments were harvested by scraping the agar using a spatula in order to obtain a concentration of 10^6^ infective particles per mL (as established using a hemocytometer). A total of 2700 seeds of each tested cereal—wheat, barley, rye, and oat—were immersed in fungal suspensions for 24 h (i.e., 900 seeds of each cereal were used per one fungal strain). Thereafter, inoculated seeds were dried on sterile filter paper in a laminar flow cabinet.

For the seed treatments, the overnight culture of the selected candidate antagonistic strain prepared in NB to a final concentration of approximately 10^8^ CFU mL^−1^ was used. To ascertain the biocontrol treatment effectiveness, sodium hypochlorite (1%)—as the most commonly used commercial seed disinfectant—was also included in the assays. The inoculated seeds were placed in Petri dishes (ø 9 cm), each containing 100 grains, representing one replicate. The inoculated seeds were treated by soaking in 10 mL of tested suspensions and were subjected to low mixing (60 rpm) for the next 18 h. Cereal seeds treated only with fungal pathogens served as positive controls. After 18 h had lapsed, treated cereal seeds were placed on a wet sterile filter paper in a plastic box to promote germination, according to the classic procedure given by the ISTA [26]. For each treatment and each fungal pathogen, a total of 300 inoculated seeds were used (100 per replicate) and the experiment was repeated twice.

The disease severity on seeds was rated using a 0–4 scale (0—seeds with no visible fungal mycelium; 1—mycelium covering up to 25% of the seed surface; 2—mycelium present on 25–50% of the seed surface; 3—mycelium covering 50–75% of the seed surface; 4—mycelium present on over 75% of the seed surface) [27]. The disease severity index (DSI) was calculated according to the following formula:

$$\mathrm{DSI}\;(\%)=\frac{\sum [\left(class\;frequency\;of\;seeds\times score\;of\;rating\;class\right)]}{\left[\left(total\;number\;of\;seeds\right)\times \left(maximal\;disease\;index\right)\right]}\;\times \;100$$

The efficacy of the applied treatments was calculated using Abbott’s formula [28]:

$$\mathrm{Efficacy}\;(\%)=[\frac{Mean\;of\;disease\;severity\;in\;control-mean\;of\;disease\;severity\;in\;treatment}{Mean\;of\;disease\;severity\;in\;control}]\;\times \;100$$

During the experiments, cereal seeds were evaluated for their germination ability in order to determine the percentage of seeds that germinated and produced normal seedlings under assay conditions. The germination rate was calculated as the average number of seeds that germinated during the assay period using the following formula:

$$\mathrm{Germination}\;(\%)=[\frac{Number\;of\;seeds\;that\;germinated}{Total\;number\;of\;seeds}]\;\times \;100$$

All data were analyzed using Statistica.Ink (version 14.0.1.25) software considering *p* < 0.01 to be statistically significant. Analysis of variance (ANOVA) was performed using Fisher’s least significant difference (LSD) tests for pairwise comparison of treatments. For correlation analyses, Pearson’s correlation coefficient was calculated to compare the DSI and germination. Principal component analysis (PCA) was performed in the statistical package Statistica.Ink (version 14.0.1.25), and the data were processed in the program R-4.3.2 and visualized in ggplot2 (version 3.5.0).

## 3. Results

### 3.1. Seedborne Fungi

**Figure 1:**
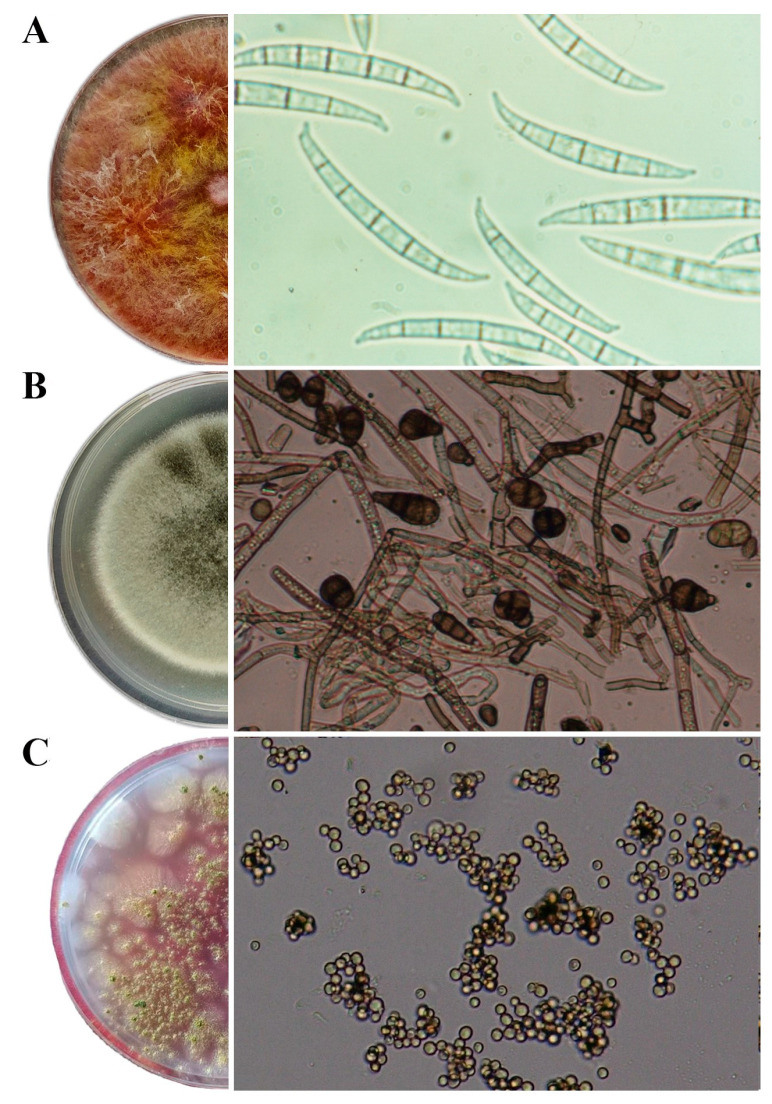
Morphological characteristics of seedborne strains: (**A**) *F. graminearum* strain WFG09, (**B**) *A. alternata* strain WAA6, and (**C**) *A. flavus* strain BFF15.

The *F. graminearum* strain WFG09 grew rapidly, with dense aerial mycelium of yellow-to-tan color, with white-to-carmine red margins. Macroconidia formed 3 to 7 septa, measured 25.8–59.2 × 2.5–5 µm in size, and were thick-walled and straight to moderately sickle-shaped. Microconidia were absent (Figure 1A). The *A. alternata* strain WAA6 formed gray, dark brown, or black fast-growing mycelia. Conidia were ellipsoidal, ovoid, and obclavate, measuring 15.2–33.8 × 7.8–13.1 μm in size. Each cell had 1–8 transverse septa and 0–2 longitudinal septa that were sometimes oblique and were brown in color (Figure 1B). The A. flavus BFF15 strain formed fast-growing yellow–green mycelia in sporulation rings. The conidia that formed in shorter unbranched chains were unicellular, rough, globose, smooth, and yellow–green in color, ranging from 3.5 to 5 µm in length (Figure 1C).

### 3.2. Identification of Selected Biocontrol Candidates

**Figure 2:**
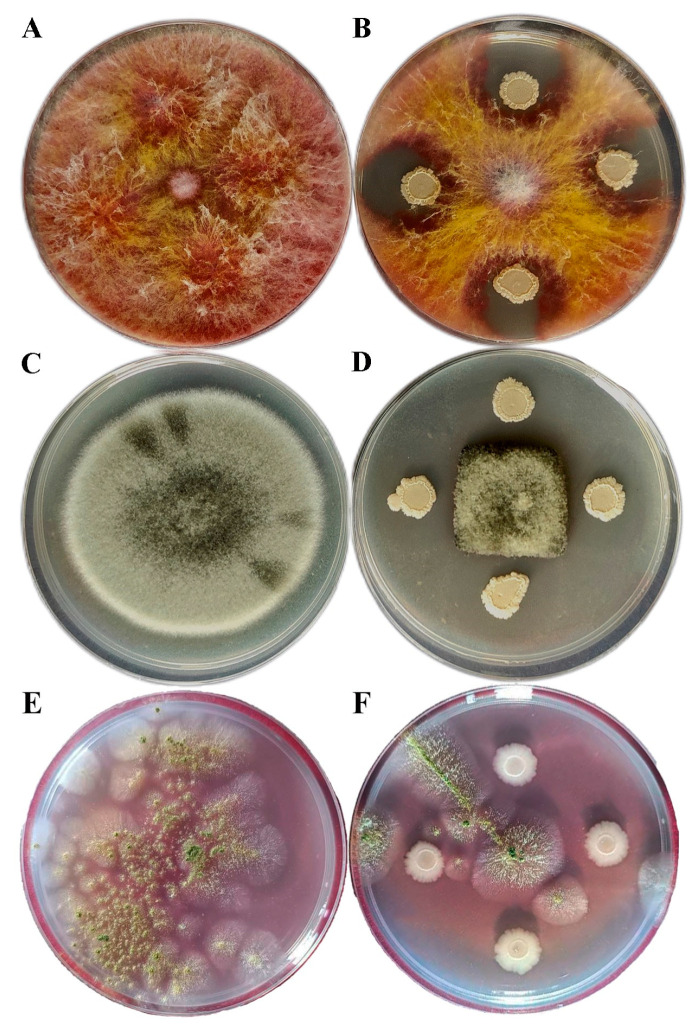
In vitro antagonistic activity of *B. halotolerans* B33 against (**B**) *F. graminearum* strain WFG09, (**D**) *A. alternata* strain WAA6, and (**F**) *A. flavus* strain BFF15 after five days of incubation, with the corresponding controls shown in (**A**), (**C**), and (**E**), respectively.

In total, 5 of the 17 tested isolates exhibited antagonistic potential, as the in vitro results demonstrated their capacity to suppress the mycelial growth of the tested fungi *F. graminearum*, *A. alternata*, and *A. flavus* (Table 2, Figure 2). The highest inhibition rate was noted for isolate B33 in all cases (54.17%, 72.09%, and 50.61% for *F. graminearum*, *A. alternata*, and *A. flavus*, respectively). Isolates B29, B30, and BML2 exhibited comparable reductions, whereas isolate B8 demonstrated the least potential in inhibiting fungal pathogens.

According to the NCBI BLASTn analysis of 16S rRNA and tuf gene sequences, isolates B29, B30, and B33 were preliminarily identified as *Bacillus halotolerans*, exhibiting 100% identity with different *B. halotolerans* strains from the NCBI (16S rRNA—strains KKD1, HFBPR26, HZ-02, SY1836, etc.; tuf—strains ZB201702, HMB20199, MBH1, and P1).

Isolate BML2 exhibited 100% similarity with *B. cereus* (16S rRNA—strain B6; tuf—SKB12), while isolate B8 was preliminarily identified as *Staphylococcus epidermidis*, showing the highest percent identity with the *S. epidermidis* strains LSAYMC, RWABASR8, AR4072, and NG02 according to the 16S rRNA gene sequence (99.83%) and strain NCCP 16828 according to the tuf gene sequence (99.86%). Given that *B. cereus* is classified under the Risk 2 biosafety level category as per the German TRBA classification [18], and since the genus *Staphylococcus* is not within the scope of beneficial organisms targeted in this study, isolates BML2 and B8 were excluded from further investigations.

**Figure 3:**
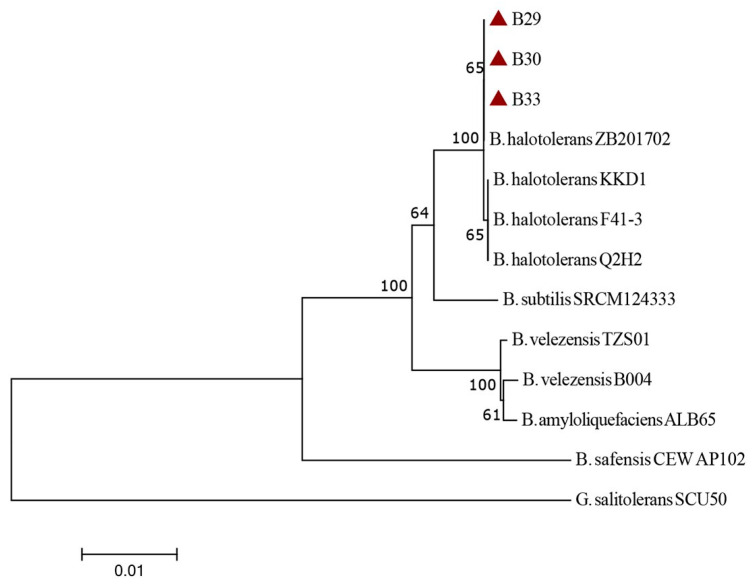
A neighbor-joining phylogenetic tree based on the concatenated sequences of the genes 16S rRNA and tuf for the three biocontrol candidate isolates (B29, B30, and B33) analyzed in this study (marked with a red triangles) and nine strains of different *Bacillus* spp. (*B. amyloliquefaciens*, *B. halotolerans*, *B. safensis*, *B. subtilis*, and *B. velezensis*) sourced from the NCBI. The tree was rooted with the G. salitolerans strain SCU50, also obtained from the NCBI.

A phylogenetic analysis performed with the concatenated sequences of both genes (16S rRNA and tuf) for the three finally selected biocontrol candidate isolates (coded as B29, B30, and B33) and nine comparative *Bacillus* spp. strains (Table 1) is presented in Figure 3. According to the obtained results, the three isolates of interest (B29, B30, and B33) were genetically homogenous. They were identified as *B. halotolerans* based on their placement within the same tree cluster as the comparative *B. halotolerans* strains ZB201702, KKD1, F41-3, and Q2H2. The remaining comparative Bacillus spp. strains (*B. amyloliquefaciens* ALB65, *B. safensis* CEW AP102, *B. subtilis* SRCM124333, and *B. velezensis* TZS01 and B004) were separated in other tree clusters, while the outgroup *G. salitolerans* strain SCU50 was placed on a monophyletic tree branch. Given that all three biocontrol candidate isolates (B29, B30, and B33) belonged to the same *Bacillus* species and were genetically homogenous according to the performed phylogenetic analysis, sequences of only one isolate (coded as B33) were deposited into the NCBI database under the accession numbers PP348669 (16S rRNA) and PP357012 (tuf).

**Figure 4:**
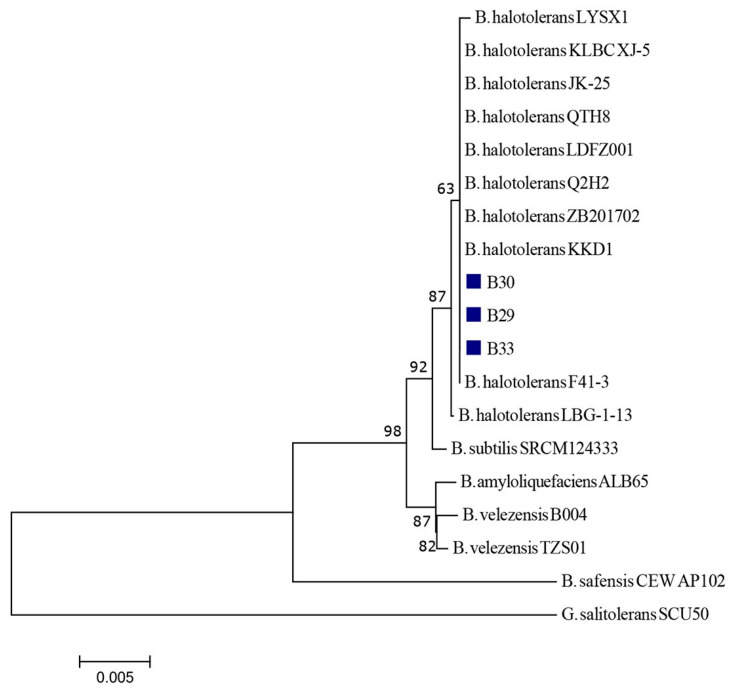
A neighbor-joining phylogenetic tree based on the 16S rRNA sequences for the three biocontrol candidate isolates (B29, B30, and B33) from this study (marked with a blue squares) and comparative strains of different *Bacillus* spp. obtained from the NCBI (*B. amyloliquefaciens*, *B. halotolerans*, *B. safensis*, *B. subtilis*, and *B. velezensis*). The tree was rooted with the *G. salitolerans* strain SCU50, sourced from the NCBI.

The second phylogenetic tree constructed with the 16S rRNA sequences is presented in Figure 4. On this tree, isolates B29, B30, and B33 are clustered with nine *B. halotolerans* comparative strains from the NCBI (ZB201702, KKD1, F41-3, Q2H2, LDFZ001, KLBC XJ-5, JK-25, LYSX1, and QTH8) demonstrating genetic homogeneity. The strain LBG-1-13 was placed in a separate subcluster (branch).

### 3.3. Biological Efficacy of the Candidate Antagonistic Strain B33

As the three *B. halotolerans* isolates B29, B30, and B33 exhibited the most pronounced in vitro antagonistic activity and were genetically homogenous according to the phylogenetic analysis, one isolate B33 presenting this group was selected for the experiment. The results of treatments against *F. graminearum*, *A. alternata*, and *A. flavus* performed on cereal seeds are given in Table 2, Table 3 and Table 4, respectively.

By comparing the DSI caused by *F. graminearum* obtained in the controls (Table 3), a statistically significant difference (*p* < 0.01) was noted for all three treatments. In the control treatment, a statistically significant difference was determined by comparing the DSI of wheat with that pertaining to barley, rye, and oat. Pairwise comparison between treatments revealed no statistically significant differences, with the exception of rye treated with the *B. halotolerans* strain B33 and sodium hypochlorite compared to all other cases. The effect of all tested treatments on germination was statistically significant compared to the controls. However, pairwise comparison between treatments revealed differences in germination rates, which were expressed in relation to the plant species used (*p* < 0.01). The obtained findings further indicated a strong negative correlation between the DSI and germination rate (*r* = −0.93, *n* = 36, *p* < 0.001), indicating that the number of germinating seeds declined as the seed contamination increased. *B. halotolerans* strain B33 was highly efficient in the control of *F. graminearum*, with an efficacy of 83.55–94.38%, comparable to that obtained with sodium hypochlorite (86.33–95.11%) as the standard (Table 3). The highest efficacy was obtained on wheat seed (94.38–95.11%), and the lowest was obtained on rye seed (83.55–86.33%).

A comparison of the DSI caused by *A. alternata* (Table 4) pertaining to the treatments with the DSI value obtained for the control treatment revealed a statistically significant difference (*p* < 0.01) in all cases. On the other hand, no statistically significant differences were found between most of the treatments, with the exception of rye treated with the *B. halotolerans* strain B33 and sodium hypochlorite, which could be clearly distinguished from other cases. Moreover, statistically significant effects of all tested treatments on germination compared to the control treatment were noted (*p* < 0.01). Further, as indicated by a strong negative correlation between the DSI and germination (*r* = −0.88, *n* = 36, *p* < 0.001), the number of germinated seeds decreased with the increase in seed contamination. The efficacy of the *B. halotolerans* strain B33 in the control of *A. alternata* ranged from 85.05 to 96.70%, which is comparable to 86.13–96.05% obtained for the sodium hypochlorite treatment (Table 4). The highest fungal control was obtained on wheat seed (96.05–96.70%), and the lowest was obtained on rye seed (85.05–86.13%).

When the DSI caused by *A. flavus* (Table 5) measured in the treatments was compared with the control values, a statistically significant difference (*p* < 0.01) was noted for all compared treatments. Conversely, with the exception of wheat treated with the *B. halotolerans* strain B33 and sodium hypochlorite, pairwise treatment comparisons yielded no statistically significant differences. Statistically significant differences, however, emerged between all tested treatments and the control with respect to germination (*p* < 0.01). According to the strong negative correlation between the DSI and germination (*r* = −0.76, *n* = 36, *p* < 0.001), seed contamination had an adverse effect on the number of germinated seeds. Finally, when applied to barley, rye, and oat seeds, the *B. halotolerans* strain B33 and sodium hypochlorite exhibited 100% efficacy in the control of A. flavus, while somewhat lower values (96.24% and 98.15%, respectively) were obtained for wheat (Table 5).

**Figure 5:**
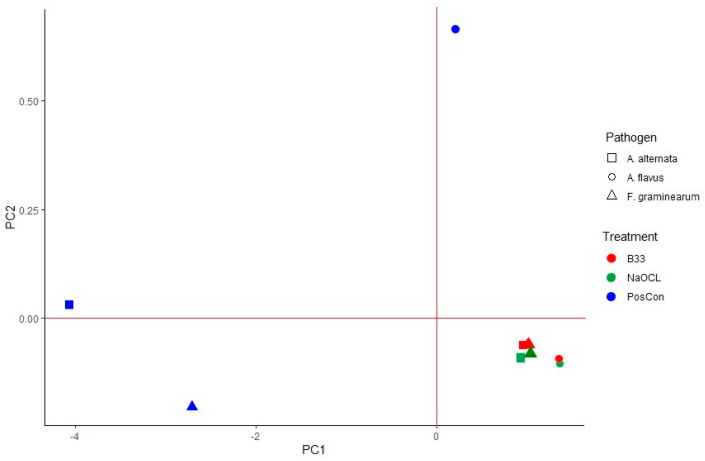
Principal component analysis (PCA) biplot (PC1 explains 98.10% and PC2 explains 1.65% of the variation in the data) with respect to the efficacy of *B. halotolerans* B33 and sodium hypochlorite treatments (indicated by shapes) for the three considered seedborne fungal pathogens—*F. graminearum*, *A. alternata*, and *A. flavus* (indicated by different colors).

When PCA was performed in order to visualize pathogen reduction in different treatments using combined data (three treatment types × three fungal pathogens), the first principal component explained 98.10% of the identified variability, while the second accounted for 1.65% of the total variance in the two principal components (PC1 and PC2) (Figure 5). According to the PCA biplot, treatments could be segregated into four groups. When all treatments and fungal pathogens were combined, it became evident that A. flavus treatments with the *B. halotolerans* strain B33 and sodium hypochlorite exhibited the highest influence. Both treatments yielded similar results when applied to *F. graminearum* and *A. alternata*. All positive control treatments were assigned to separate subclusters. In general, according to their antagonistic activity, the *B. halotolerans* strain B33 and sodium hypochlorite (a commercial product used as a standard pathogen control measure) exhibited the same efficacy as the seed treatments against the three tested fungal pathogens (*F. graminearum*, *A. alternata*, and *A. flavus*).

## 4. Discussion

In this study, the *B. halotolerans* strain B33, isolated from soil at a rural, non-agricultural site, was identified as a potential biocontrol agent against seedborne polyphagous fungal pathogens (*F. graminearum*, *A. alternata*, and *A. flavus*) on wheat, barley, oat, and rye as test hosts, showing a high efficacy rate on the seed of all four cereals. In addition to its biocontrol potential, treatment with this strain enhanced the seed germination rate. Bacterium *B. halotolerans* recently gained the attention of the research community due to its plant growth-promoting and biocontrol abilities. Although *B. halotolerans* was mostly known for its use as an abiotic stress mitigation agent, and its potential to increase tolerance to drought and salinity stress was experimentally demonstrated in [22,29,30], research on its potential use against biotic stress is limited and mostly recent [19,20,21,22,23,24,25,31,32]. In the extant literature, several *B. halotolerans* strains with antagonistic activity against various fungal pathogens are described, namely LDFZ001 [21], KLBC XJ-5 [20], Jk-25 [24], Q2H2 [25], LBG-1-13 [22], RFP74 [32], BFOA1–BFOA4 [31], QTH8 [23], and Hil4 [14]. Phylogenetic analysis based on 16S rRNA performed with the three *Bacillus* isolates (B29, B30, and B33) in focus in the current investigation demonstrated their genetic homogeneity with most of the aforementioned *B. halotolerans* strains (Q2H2, LDFZ001, KLBC XJ-5, Jk-25, and QTH8), while strain LBG-1-13 was separated in another subcluster. These results indicate genetic homogeneity among different *B. halotolerans* strains regardless of their origin.

Our analyses revealed that the *B. halotolerans* strain B33 is highly effective in the control of *F. graminearum*, achieving up to 94.38% efficacy depending on the cereal type. Based on the calculated disease severity index of cereal seeds infected by *A. alternata*, this strain was up to 96.70% effective in the control of this pathogen. In both cases, the highest efficacy was obtained on wheat, while the lowest was noted on rye seed. Moreover, this is a pioneering study describing the activity of *B. halotolerans* against the fungus *A. flavus*, with a rated efficacy of 100% on barley, rye, and oat seeds, while its efficacy on wheat was slightly lower at 96.24%. Similarly, as a part of their study, Kang et al. [24] showed significant biocontrol effects of strain Jk-25 against *Fusarium oxysporum* and *F. graminearum*, with inhibition rates of 80.47% and 75.33%, respectively. Moreover, Wang et al. [25] recently reported strong antagonism of strain Q2H2 (isolated from the potato plant roots) against *Fusarium* fungi (*F. oxysporum*, *F. commune*, *F. graminearum*, and *F. brachygibbosum*). Strain LBG-1-13 described by Gao et al. [22] has antagonistic activity against the lily pathogen *F. oxysporum*, as well as against the fungi *Botrytis cinerea* and *Botryosphaeria dothidea*. Four bacterial isolates, designated as BFOA1–BFOA4 by Slama et al. [31], proved to be very active against *Fusarium* isolates belonging to the species *F. oxysporum*, *F. solani*, *F. acuminatum*, and *F. chlamydosporum*, as well as against the phytopathogenic fungi *B. cinerea*, *A. alternata*, *Phytophthora infestans*, and *Rhizoctonia bataticola*. According to Rafanomezantsoa et al. [32], the strain RFP74 inhibited the growth of various phytopathogens, including *Alternaria* (*A. tomatophila*, *A. alternata*, and *A. solani*) in vitro, with an inhibition rate exceeding 60%. Similarly, in this study, we obtained an inhibition rate of 72.09% for the *B. halotolerans* strain B33 against the fungus *A. alternata*.

A wider domain of antimicrobial activity for *B. halotolerans* was established by Li et al. [23] using culture filtrates of the bacterial strain QTH8, which inhibited the mycelial growth of various fungal species—*F. graminearum*, *F. pseudograminearum*, *Hainesia lythri*, *Pestalotiopsis* sp., *B. cinerea*, *Curvularia lunata*, *Phyllosticta theaefolia*, *Phytophthora nicotianae*, and *Sclerotinia sclerotiorum*. Based on earlier investigations, Xia et al. [19] highlighted the *B. halotolerans* strain LYSX1 as a potential microbe for the sustainable biocontrol of root-knot nematodes through induced systemic resistance in tomato. According to the gene editing experiments conducted by Feng et al. [21], functional expression of phosphopantetheinyl transferase and major facilitator superfamily transporter genes in *B. halotolerans* LDFZ001 is essential for its antifungal activity against the pathogen *Rhizoctonia solani*. Similarly, based on their study of the biocontrol ability and action mechanisms of the *B. halotolerans* strain KLBC XJ-5 in the control of gray mold disease (*B. cinerea*) in postharvest strawberries, Wang et al. [19] concluded that this strain controlled mycelial growth as well as conidial germination in vitro. They further noted that the strain KLBC XJ-5 harbored six antimicrobial biosynthesis gene clusters and four glycoside hydrolase family members involved in chitin degradation. Whole genome sequencing of the *B. halotolerans* endophytic bacterial strain Hil4 (isolated from leaves of the medicinal plant *Hypericum hircinum*) revealed that it possesses numerous secondary metabolite biosynthetic gene clusters and genes involved in plant growth promotion, colonization, and plant defense elicitation [14].

In the current study, we found that *B. halotolerans* B33 treatment had discernible effects on seed germination quality, indicating its potential for use in plant growth promotion. Hence, the importance of this research lies in its pioneering approach to seed treatment targeting seedborne fungal pathogens, ultimately enhancing the quality of germination through such application. The biocontrol solution obtained in this work may have practical application in sustainable agriculture as it can be adopted in the management of multiple pathogens, not just on small-grained cereals that served as a plant model system in this work but also on a broader host range of the tested pathogens. Therefore, the present study sheds light on an environmentally friendly alternative to the conventional use of chemical substances in the control of seedborne pathogens. Further studies with *B. halotolerans* B33 need to be carried out under the greenhouse and open field conditions to validate the real-world applicability of the obtained findings, as well as assess the degree of potential variability under different environmental conditions. Despite its limitations—the application being performed only on seeds and the lack of understanding of the mechanisms underlying the biocontrol efficacy of the *B. halotolerans* strain B33—this research enriches the current knowledge of *B. halotolerans* and its application in biocontrol, offering a foundation for further investigations into sustainable cereal seed treatment strategies.

## 5. Conclusions

This research highlights the promising biocontrol capabilities of the *B. halotolerans* strain B33, both in in vitro assays and seed treatment experiments, against seedborne fungal pathogens—*F. graminearum*, *A. alternata*, and *A. flavus*—on small cereal seeds. By investigating its activity across multiple plant pathogens and crops, the investigations carried out as a part of this work provide a valuable contribution to sustainable agriculture, particularly as the application of this strain to cereals has not been studied previously. The demonstrated efficacy suggests that this is a promising environmentally friendly alternative to the conventional synthetic fungicides commonly used for such purposes. Moreover, control practices based on potent biological agents could ensure a sustainable supply of healthy cereals free of pesticide residues as well as toxins produced by some seedborne fungi.

## Figures and Tables

**Table 1:** A list of *Bacillus* spp. strains from the GenBank database used for NJ phylogenetic analysis.

Species	Strain	Isolation Source	Country	Accession No.
*B. amyloliquefaciens*	ALB65	Alfalfa silage	USA	CP029069
*B. halotolerans*	KKD1	Saline–alkali rhizosphere soil	China	CP054584
	F41-3	Flower of Chinese redbud	South Korea	CP041357
	ZB201702	Rhizosphere soil of maize	China	CP029364
	Q2H2	Potato root endophyte	China	CP136430
	LDFZ001	Soil	China	CP063276
	LYSX1	Rhizosphere soil of tobacco	China	MH359399
	QTH8	Rhizosphere soil of *Cotinus coggygria*	China	MN410608
	JK-25	Wheat soil	China	OP804109
	KLBC XJ-5	Strawberry fruit	China	MN853407
	LBG-1-13	Root of lily	China	MW199064
*B. safensis*	CEW AP102	Marine or sediment	India	CP126090
*B. subtilis*	SRCM124333	Gochujang	South Korea	CP116012
*B. velezensis*	B004	Citrus leaf	China	CP141895
	TZS01	Sinkhole	Mexico	CP141828

**Table 2:** Cereal seedborne pathogen inhibition rates of effective antagonistic bacterial isolates.

Antagonistic Isolate	Inhibition Rate (%)		
	Isolate WFG09 (*F. graminearum*)	Isolate WAA6 (*A. alternata*)	Isolate BFF15 (*A. flavus*)
B29	43.33	62.79	48.27
B30	52.50	55.81	45.67
B33	54.17	72.09	50.61
BML2	48.06	53.83	48.32
B8	28.53	22.91	25.93

**Table 3:** Efficacy of *B. halotolerans* strain B33 on treated cereal seeds infected by *Fusarium graminearum*.

Plant Species	Treatment								
	*Bacillus halotolerans* Strain B33			Sodium Hypochlorite (1%)			Control		
	Disease Index (%)	Efficacy (%)	Germination (%)	Disease Index (%)	Efficacy (%)	Germination (%)	Disease Index (%)	Efficacy (%)	Germination (%)
Wheat	1.92 ± 1.18 ^1^a^2^(a)^3^	94.38	87.33 ± 3.20 ^1^a^2^	1.67 ± 0.80 ^1^a^2^(a)^3^	95.11	87.33 ± 5.03 ^1^a^2^	34.17 ± 3.82 ^1^c^2^	-	61.33 ± 6.82 ^1^e^2^
Barley	3.00 ± 1.56 ^1^ab^2^(a)^3^	92.47	83.67 ± 0.58 ^1^ab^2^	3.00 ± 1.73 ^1^ab^2^(a)^3^	92.47	77.00 ± 7.00 ^1^cd^2^	39.83 ± 5.79 ^1^d^2^	-	52.00 ± 5.65 ^1^f^2^
Rye	6.92 ± 0.63 ^1^b^2^(b)^3^	83.55	75.67 ± 4.93 ^1^d^2^	5.75 ± 0.66 ^1^ab^2^(b)^3^	86.33	79.66 ± 2.52 ^1^bcd^2^	42.08 ± 5.20 ^1^d^2^	-	51.67 ± 7.23 ^1^f^2^
Oat	2.92 ± 1.66 ^1^ab^2^(a)^3^	93.16	81.67 ± 3.79 ^1^abcd^2^	2.50 ± 0.87 ^1^a^2^(a)^3^	94.14	82.33 ± 2.52 ^1^abc^2^	42.67 ± 3.50 ^1^d^2^	-	51.00 ± 2.65 ^1^f^2^

^1^ Average disease severity index (DSI) value ± standard deviation; ^2^ a statistical relationship between treatments (Fisher’s LSD tests, *p* < 0.01) is marked with letters, whereby different letters indicate the existence of statistical significance; ^3^ a statistical relationship between treatments (Fisher’s LSD tests, *p* < 0.01) excluding the control treatment, where different letters indicate the existence of statistical significance.

**Table 4:** Efficacy of *B. halotolerans* strain B33 on treated cereal seeds infected by *Alternaria alternata*.

Plant Species	Treatment								
	*Bacillus halotolerans* Strain B33			Sodium Hypochlorite (1%)			Control		
	Disease Index (%)	Efficacy (%)	Germination (%)	Disease Index (%)	Efficacy (%)	Germination (%)	Disease Index (%)	Efficacy (%)	Germination (%)
Wheat	1.67 ± 0.80 ^1^a^2^(a)^3^	96.70	83.33 ± 2.89 ^1^a^2^	2.00 ± 0.50 ^1^a^2^(a)^3^	96.05	81.33 ± 3.51 ^1^ab^2^	50.67 ± 8.41 ^1^b^2^	-	50.33 ± 5.13 ^1^c^2^
Barley	3.25 ± 1.75 ^1^a^2^(a)^3^	93.87	76.33 ± 1.53 ^1^ab^2^	3.75 ± 2.38 ^1^a^2^(a)^3^	92.92	73.67 ± 4.73 ^1^ab^2^	53.0 ± 3.70 ^1^b^2^	-	49.33 ± 3.21 ^1^c^2^
Rye	8.17 ± 1.70 ^1^a^2^(b)^3^	85.05	74.33 ± 8.14 ^1^ab^2^	7.58 ± 1.23 ^1^a^2^(b)^3^	86.13	74.00 ± 6.93 ^1^ab^2^	54.67 ± 11.54 ^1^b^2^	-	56.67 ± 6.11 ^1^c^2^
Oat	3.25 ± 0.25 ^1^a^2^(a)^3^	93.84	79.33 ± 5.86 ^1^ab^2^	3.92 ± 1.66 ^1^a^2^(a)^3^	92.57	72.67 ± 6.43 ^1^ab^2^	52.75 ± 10.01 ^1^b^2^	-	55.00 ± 12.00 ^1^c^2^

^1^ Average disease severity index (DSI) value ± standard deviation; ^2^ a statistical relationship between treatments (Fisher’s LSD tests, *p* < 0.01) is marked with letters, whereby different letters indicate the existence of statistical significance; ^3^ a statistical relationship between treatments (Fisher’s LSD tests, *p* < 0.01) excluding the control treatment, where different letters indicate the existence of statistical significance.

**Table 5:** Efficacy of *B. halotolerans* strain B33 on treated cereal seeds infected by *Aspergillus flavus*.

Plant Species	Treatment								
	*Bacillus halotolerans* Strain B33			Sodium Hypochlorite (1%)			Control		
	Disease Index (%)	Efficacy (%)	Germination (%)	Disease Index (%)	Efficacy (%)	Germination (%)	Disease Index (%)	Efficacy (%)	Germination (%)
Wheat	0.67 ± 1.15 ^1^a^2^(bc)^3^	96.24	90.33 ± 3.06 ^1^a^2^	0.33 ± 0.58 ^1^a^2^(b)^3^	98.15	89.00 ± 8.72 ^1^a^2^	17.83 ± 2.96 ^1^d^2^	-	70.67 ± 5.13 ^1^c^2^
Barley	0 ^1^a^2^(a)^3^	100.00	88.67 ± 1.53 ^1^a^2^	0 ^1^a^2^(a)^3^	100.00	91.67 ± 4.73 ^1^a^2^	5.25 ± 1.32 ^1^c^2^	-	71.67 ± 10.12 ^1^bc^2^
Rye	0 ^1^a^2^(a)^3^	100.00	89.00 ± 8.72 ^1^a^2^	0 ^1^a^2^(a)^3^	100.00	88.67 ± 1.53 ^1^a^2^	19.17 ± 1.44 ^1^d^2^	-	69.00 ± 12.53 ^1^c^2^
Oat	0 ^1^a^2^(a)^3^	100.00	92.33 ± 2.08 ^1^a^2^	0 ^1^a^2^(a)^3^	100.00	91.67 ± 4.73 ^1^a^2^	2.42 ± 2.04 ^1^b^2^	-	79.67 ± 6.66 ^1^b^2^

^1^ Average disease severity index (DSI) value ± standard deviation; ^2^ a statistical relationship between treatments (Fisher’s LSD tests, *p* < 0.01) is marked with letters, whereby different letters indicate the existence of statistical significance; ^3^ statistical relationship between treatments (Fisher’s LSD tests, *p* < 0.01) excluding the control treatment, where different letters indicate the existence of statistical significance.

## Data Availability

All relevant data are within the article.
